# Iron parameters in patients with end-stage renal disease receiving lanthanum carbonate or other non-iron-based phosphate binders: Results from a phase 3, randomized open-label study

**DOI:** 10.1177/2050312118786161

**Published:** 2018-07-02

**Authors:** Rosamund J Wilson, Beverly Jones, Claudio Marelli

**Affiliations:** 1 Spica Consultants Limited, Marlborough, UK; 2 Shire, Lexington, MA, USA; 3 Shire, Zug, Switzerland

**Keywords:** End-stage renal disease, hyperphosphataemia, iron parameters, lanthanum carbonate, phosphate binder

## Abstract

**Objectives:**

The recent availability of iron-based phosphate binders has raised some concerns about iron overload in patients with end-stage renal disease. This study evaluated iron parameters in patients with end-stage renal disease receiving lanthanum carbonate or other non-iron-based phosphate binders.

**Methods:**

This analysis used 2-year follow-up data from an open-label, multicentre, randomized, active-controlled, parallel-group, phase 3 trial of lanthanum carbonate (SPD405-307). After a washout period, if patients’ serum phosphate levels exceeded 5.9 mg/dL, they were randomized 1:1 to receive lanthanum carbonate (375–3000 mg/day) or non-iron-based standard therapy during a 6-week dose titration period. Patients achieving control of serum phosphate levels (⩽5.9 mg/dL) received maintenance therapy with lanthanum carbonate or standard therapy for up to 24 months.

**Results:**

No clinically relevant changes in mean (standard deviation) iron parameters between the treatment groups (lanthanum carbonate, n = 682; standard therapy, n = 677) from baseline to month 24/final visit were observed: iron (µg/dL), −1.1 (41.8) versus 1.0 (38.7); ferritin (ng/mL), 208.4 (445.1) versus 262.4 (505.5); transferrin saturation (%), 2.8 (18.0) versus 2.8 (17.3); and haemoglobin (g/dL), 0.4 (1.9) versus 0.3 (1.7), respectively (all, p > 0.1). There were no clinically relevant changes in the percentage of patients receiving any anti-anaemic preparation in either treatment group (pre- vs post-randomization: lanthanum carbonate, 94.9% vs 97.8%; standard therapy, 95.1% vs 98.8%, respectively). This is in contrast to the study by Lewis and colleagues, which found significant increases in ferritin and transferrin saturation levels in patients receiving ferric citrate versus active control (calcium acetate and/or sevelamer carbonate) after 52 weeks of therapy. Although serum ferritin and transferrin saturation are the recommended iron indices by the Kidney Disease Outcome Quality Initiative, they are indirect indicators of iron status. Longer-term studies are required to understand fully the potential risks associated with iron overload.

**Conclusion:**

No evidence of iron accumulation was found in patients with end-stage renal disease receiving lanthanum carbonate or other non-iron-based binders.

## Introduction

Patients with end-stage renal disease (ESRD) frequently develop a range of comorbidities, including anaemia and hyperphosphataemia.^1^ Iron supplements and blood transfusions have been used to treat anaemia in patients with ESRD.^2^ In these patients, iron neutrality is the desired outcome, and any need for iron supplementation should be addressed with appropriate treatments for each patient.^3^ Iron overload can occur and may be associated with increased morbidity and mortality,^2,4^ although, since the availability of recombinant human erythropoietin therapy, the incidence of iron overload has decreased.^2^

**Figure 1.**
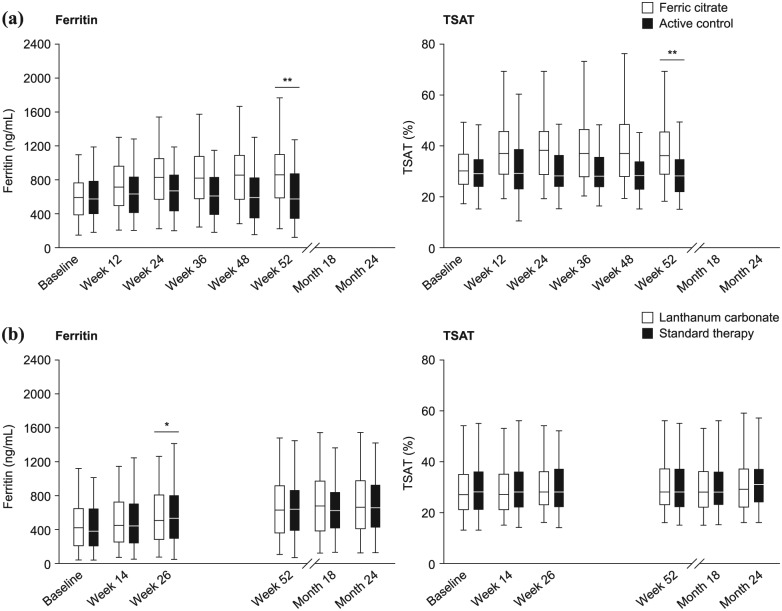
Changes in ferritin and TSAT levels over (a) 52 weeks of treatment with ferric citrate^9^ and (b) 24 months of treatment with lanthanum carbonate (study SPD405-307). Source: Panel (a) adapted with permission from the *Journal of the American Society of Nephrology* by the American Society of Nephrology, from Lewis et al.^9^ Copyright^©^ 2015; permission conveyed through the Copyright Clearance Centre. TSAT: transferrin saturation. Data are presented as the 5th, 25th, median, 75th and 95th percentiles. (a) All p values were only available for 52-week comparisons between the treatment arms (**p < 0.001). (b) All post-baseline comparisons between the treatment arms were non-significant (p > 0.05), except for serum ferritin levels at week 26 (*p = 0.004).

Patients with hyperphosphataemia are treated with phosphate binders to lower serum phosphate levels;^1^ however, the recent availability of iron-based binders (sucroferric oxyhydroxide (PA21)^5^ and ferric citrate^6^) has raised new concerns about the potential of iron overload.^7^ Iron uptake from PA21 has been shown to be low,^8^ so iron overload is not considered to be a safety concern with this binder.^5^ In contrast, patients with ESRD receiving ferric citrate had higher mean (±standard deviation (SD)) iron parameters after 52 weeks of treatment than individuals receiving active control (ferritin levels, 899 ± 488 vs 628 ± 367 ng/mL, p < 0.001; transferrin saturation (TSAT), 39% ± 17% vs 30% ± 12%, p < 0.001; Figure 1(a)).^9^ This is probably due to gastrointestinal absorption of iron from ferric citrate. Based on the recent availability of these data, it is now pertinent that the corresponding previously unpublished data for lanthanum carbonate (LaC; a non-iron-based phosphate binder) be made available for comparison.

The aim of this analysis was therefore to evaluate iron parameters in patients with ESRD receiving LaC and other non-iron-based phosphate binders using follow-up data from a 2-year active-controlled, phase 3 trial of LaC (SPD405-307).^10^ An indirect comparison of published data to assess tablet burden considerations for ferric citrate, LaC and sevelamer carbonate/hydrochloride was also performed.

## Methods

### Study design and participants

SPD405-307 was a phase 3, open-label, multicentre, randomized, active-comparator-controlled, parallel-group trial of LaC.^10^ The trial comprised 110 study sites from the United States, Puerto Rico, Poland and South Africa. The primary objective was to assess the long-term efficacy and safety of LaC compared with standard phosphate binder therapy (non-iron-based, standard therapy (ST)) in patients with ESRD. The study began with a washout period (1−3 weeks) to allow patients’ serum phosphate levels to return to pre-treatment values. If serum phosphate levels exceeded 5.9 mg/dL at the end of washout, patients were randomized 1:1 to receive either LaC (375−3000 mg/day) or ST during a 6-week dose titration period. An interactive voice response system was used to assign patients randomly into one of the two treatment arms, and block randomization with a block size of four was employed to generate the treatment assignment schedule. Patients achieving control of serum phosphate levels (⩽5.9 mg/dL) at the end of dose titration entered a treatment period, during which they received a maintenance dose (determined by dose titration) of LaC or ST for up to 24 months.

The study was conducted in accordance with the ethical principles set out in the Declaration of Helsinki. The protocol was approved at each centre by an Institutional Review Board, and the patients provided written, informed consent before participation.

### Key inclusion criteria

Patients (⩾12 years old) with ESRD were included if they had been receiving haemodialysis three times weekly for the 2 months before the start of the study and had hyperphosphataemia (serum phosphate levels > 5.9 mg/dL).

### Iron parameters

Clinical laboratory tests (including iron parameters) were pre-specified safety assessments that were monitored for the duration of the trial. Patients’ iron (µg/mL), ferritin (ng/mL), TSAT (%) and haemoglobin (g/dL) levels were measured at baseline and post-treatment (month 24/final visit). Levels were also measured at set intervals during the maintenance period (weeks 14, 26 and 52, and months 18 and 24).

### Concomitant medication usage

Patient data were analysed in order to assess the percentage of patients taking concomitant medications (specifically anti-anaemic preparations) pre- and post-randomization. As some medication dates were partially recorded or missing, the following rules were applied.

#### Concomitant medication start dates

If the year was missing, the year remained as missing, and no imputation was performed. If the year was recorded, but the month and day were missing, 1 January of that year was used as the start date. If the day was missing, the first day of that month was used as the start date.

#### Concomitant medication end dates

If the year was missing, the year remained as missing, and no imputation was performed. If the year was recorded, but the month and day were missing, 31 December was used as the end date. If the day was missing, the last day of that month was used as the end date.

‘Pre-’ and ‘post-’ randomization categories were then created based on the following rules (rules were as conservative as possible):

1. If the medication end date was before the randomization date, the medication was considered to be ‘pre’.
2. If the medication start date was before the randomization date, and the medication end date was missing, the medication was considered to be both ‘pre’ and ‘post’.
3. If the medication start date was before the randomization date, and no end date was recorded, but it was noted that the patient continued to take the medication, then the medication was considered to be both ‘pre’ and ‘post’.
4. If the medication start date was before the randomization date, and the medication end date was on or after the randomization date, the medication was considered to be both ‘pre’ and ‘post’.
5. If the concomitant medication start date was missing, and the medication end date was missing, the medication was considered to be both ‘pre’ and ‘post’.
6. If the concomitant medication start date was missing, and no end date was recorded, but it was noted that the patient continued to take the medication, then the medication was considered to be both ‘pre’ and ‘post’.
7. If either the medication start date or the medication end date was on or after the randomization date, the medication was considered to be ‘post’.

### Statistical analysis

All statistical analyses were performed in SAS^®^ version 9.4 (SAS Institute, Cary, NC, USA). The primary goal of this study was to assess safety, and as such no statistical estimate for sample size was performed.

#### Iron parameters

A mixed-effects model for repeated-measures data was used for the analysis of changes in iron parameters. The model included fixed effects for treatment, visit, treatment-by-visit interaction, a random effect for patient and other covariates including baseline results. The treatment, visit and treatment-by-visit interaction were included in the final model.

#### Concomitant medication usage

Statistical comparisons of concomitant medication usage were performed post hoc only to evaluate the increase in ferritin levels observed in both the treatment arms over the 24-month period.

Given the number of possible comparisons here, statistically significant differences were expected to be observed by chance, even for changes that would not be considered clinically relevant. On medical review, a 10% difference in the percentage of patients either between the treatment arms or between pre- and post-randomization was considered clinically important. A 10% difference in patients, either for between-treatment-arm comparisons or for between-pre- and post-randomization comparisons, was therefore used as a cutoff for statistical testing; p values (from Pearson’s chi-square tests) or 95% confidence intervals (CIs) were subsequently only presented where a 10% difference in the percentage of patients for a parameter was observed.

### Indirect comparison of ferric citrate and LaC

Data from two studies were used to compare phosphate levels and tablet burden considerations indirectly in patients receiving either ferric citrate or LaC. These were a study by Wilson et al.,^11^ which compared the efficacy of LaC and sevelamer hydrochloride in patients with ESRD after 16 weeks (950 patients were included in this analysis), and a randomized clinical trial by Lewis et al.,^9^ which compared the efficacy of ferric citrate with sevelamer carbonate in patients with ESRD after 52 weeks (441 patients were included in this analysis). The bioequivalence of sevelamer carbonate and sevelamer hydrochloride has been confirmed, and so the anticipated doses required to achieve similar phosphate levels were considered to be equal.^12^ At the end of the titration period, phosphate levels in the treatment arms (LaC or ferric citrate compared with sevelamer) were similar.^9,11^ The doses of LaC, ferric citrate and sevelamer used in these studies could therefore be compared to assess pill burden. This indirect comparison was performed to determine the relative doses of ferric citrate and LaC required to achieve similar serum phosphate levels, because a high dose burden could potentially affect iron parameters in patients receiving ferric citrate. These comparisons assumed the following: LaC (1000-mg tablet), sevelamer carbonate (800-mg tablet) and sevelamer hydrochloride (800-mg tablet).

## Results

### Patient demographics

**Figure 2.**
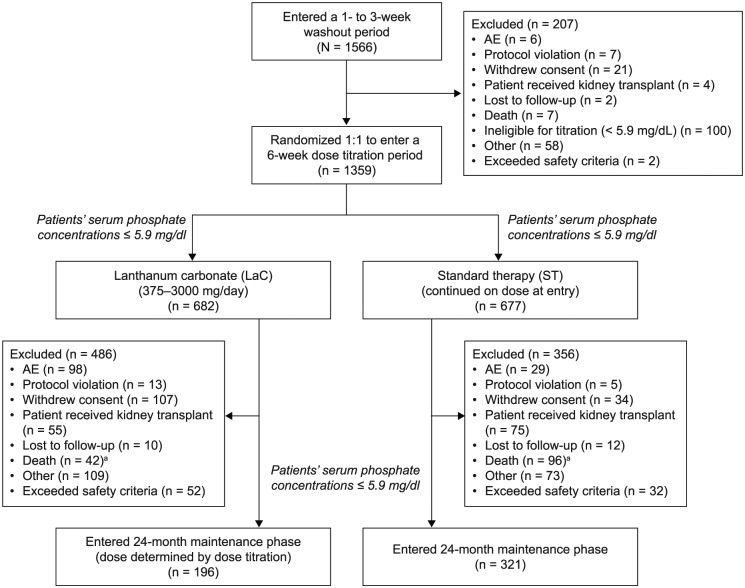
Patient flow diagram. LaC: lanthanum carbonate; ST: standard therapy. ^a^Patients who died after study termination (LaC, n = 13; ST, n = 19).

A patient flow diagram is presented in Figure 2. A total of 1566 patients entered the 1- to 3-week washout period. Of these, 1359 patients were randomized 1:1 (LaC, n = 682; ST, n = 677). A total of 517 patients completed the study (LaC, n = 196; ST, n = 321). Reasons for discontinuation are presented in Figure 2. Patient characteristics are reported elsewhere.^10^ In brief, patient characteristics were not statistically different between the treatment arms (LaC or ST) at baseline: mean (SD) age – 53.8 (14.6) versus 54.9 (14.4) years, p = 0.141; height – 169.7 (11.2) versus 170.7 (10.9) cm, p = 0.105; and weight – 80.7 (21.8) versus 81.0 (21.3) kg, p = 0.751, respectively. Patients receiving ST continued with their treatment at entry to the study (calcium-based binders, 78.2%; sevelamer, 15.8%; and other/not captured, 5.9%).

### Iron parameters

There were no clinically relevant or statistically significant differences in changes in mean (SD) iron parameters from the baseline to the final visit between the treatment groups (LaC or ST): iron (µg/dL), −1.1 (41.8) versus 1.0 (38.7), p = 0.558; ferritin (ng/mL), 208.4 (445.1) versus 262.4 (505.5), p = 0.220; TSAT (%), 2.8 (18.0) versus 2.8 (17.3), p = 0.996; and haemoglobin (g/dL), 0.4 (1.9) versus 0.3 (1.7), p = 0.783 (Table 1). In addition, there were no clinically relevant differences in ferritin or TSAT levels between patients receiving LaC or ST at any time point post-dose titration during the study (Figure 1(b)); all of these comparisons were non-significant (p > 0.05), except for serum ferritin at week 26, where a statistically significant difference was observed between LaC and ST (p = 0.004). This was due to an observed larger increase in the ST group than in LaC, but was a one-off difference not seen at future visits.

**Table 1.** Changes in iron parameters and haemoglobin levels from baseline for patients receiving lanthanum carbonate and standard therapy (study SPD405-307)^a^.

Parameter	Treatment arm		p value*
	Lanthanum carbonate	Standard therapy	
Iron (µg/dL)	−1.1 ± 41.8 (n = 195)	1.0 ± 38.7 (n = 320)	0.558
Ferritin (ng/mL)	208.4 ± 445.1 (n = 195)	262.4 ± 505.5 (n = 319)	0.220
Transferrin saturation (%)	2.8 ± 18.0 (n = 183)	2.8 ± 17.3 (n = 303)	0.996
Haemoglobin (g/dL)	0.4 ± 1.9 (n = 189)	0.3 ± 1.7 (n = 294)	0.783

a Changes from baseline to month 24/final visit. Data are presented as the change from baseline (mean ± standard deviation).

* p values represent the comparisons between the treatment arms from a mixed-effects model including fixed effects for treatment, visit, treatment-by-visit interaction, a random effect for patient and other covariates including baseline results. The treatment, visit and treatment-by-visit interaction were included in the final model.

For both the treatment groups, there was a general increase in serum ferritin levels over time (Figure 1(b)). Although this does not appear to be consistently different between the treatment arms, post hoc statistical testing was performed to evaluate whether these increases were statistically different from baseline in each treatment arm. A paired t-test showed that increases in serum ferritin levels from baseline to month 24 were statistically significant in both the treatment arms (p < 0.001).

A higher proportion of patients in this study were receiving intravenous iron post-randomization, likely since they were seeing their nephrologist more regularly as part of this study, which could potentially explain the increase in serum ferritin observed from the baseline to month 24/final visit in both the treatment arms (Table 1).

### Concomitant medication usage

The changes in the percentage of patients receiving any anti-anaemic preparation from pre- to post-randomization were not considered clinically meaningful for either treatment group (LaC, 94.9% vs 97.8%; ST, 95.1% vs 98.8%). The percentage of patients using intravenous anti-anaemic preparations or other intravenous anti-anaemic preparations (non-iron-based) increased post-randomization in both the treatment groups (by up to 12% in each group). There were no differences pre-randomization in the percentage of patients using intravenous iron between the treatment arms (p = 0.373); however, post-randomization, more patients on ST were receiving intravenous iron than patients on LaC (p < 0.0001). Both the treatment arms showed increases in ferritin levels, and this was numerically greater in the ST arm; this result is consistent with the hypothesis that this may be driven by the use of intravenous iron medications. The 95% CIs for pre- and post-randomization values for LaC (43.3%–50.8% pre-randomization and 69.5%–76.2% post-randomization) indicate a notable statistical difference between the results, as the CIs do not overlap. The same is true for ST, where the CIs for the pre- and post-randomization values for ST were 45.7%–53.2% pre-randomization and 81.0%–86.5% post-randomization (Table 2).

**Table 2.** Concomitant medications (anti-anaemic preparations) taken by patients in each treatment group in the SPD405-307 study, pre-randomization and post-randomization.

Concomitant medication	Lanthanum carbonate (n = 682)	Standard therapy (n = 677)	p value*
Anti-anaemic preparations (all)^a^, n (%)			
Pre-randomization	647 (94.9)	644 (95.1)	–
Post-randomization	667 (97.8)	669 (98.8)	–
Anti-anaemic preparations (intravenous)^b^, n (%)			
Pre-randomization	578 (84.8)	575 (84.9)	–
Post-randomization	637 (93.4)	642 (94.8)	–
Iron preparations (intravenous)^c^, n (%) [95% CI]*			
Pre-randomization	321 (47.1) [43.3–50.8]	335 (49.5) [45.7–53.2]	0.373
Post-randomization	497 (72.9) [69.5–76.2]	567 (83.8) [81.0–86.5]	<0.0001
Other anti-anaemic preparations (intravenous)^d^, n (%)			
Pre-randomization	511 (74.9)	522 (77.1)	–
Post-randomization	574 (84.2)	601 (88.8)	–

CI: confidence interval.

a Anti-anaemic preparations (all) include the terms listed below (^a–c^) and also B03AA (ferro-sequels, ferrous phosphogluconate, Vitron-C); B03AB (ferritin, polysaccharide–iron complex); B03AD (iron in combination with folic acid, ferro-folsan, ferrous sulphate, pregamal); B03AE (chromagen, galenic/iron/vitamins (nitric oxide supplements)/folic acid, iberet-folic) and B03XA (other anti-anaemic preparations). Route of administration not reported here.

b Anti-anaemic preparations (intravenous) include the terms B03A (ferric sodium gluconate complex, iron, iron preparations); B03AA (ferrous fumarate, ferrous gluconate, ferrous sulphate); B03AB (saccharated iron oxide); B03AC (sodium ferric gluconate complex); B03AE (iron dextran, prenatal); B03BA (cyanocobalamin); B03BB (folic acid) and B03XA (epoetin alfa, erythropoietin, erythropoietin human).

c Iron preparations (intravenous) include the terms B03A (ferric sodium gluconate complex, iron, iron preparations); B03AA (ferrous fumarate, ferrous gluconate, ferrous sulphate); B03AB (saccharated iron oxide); B03AC (sodium ferric gluconate complex); B03AE (iron dextran, prenatal); B03BA (cyanocobalamin) and B03BB (folic acid).

d Other anti-anaemic preparations (intravenous) include the term B03XA (epoetin alfa, erythropoietin, erythropoietin human).

* p values represent the comparisons between the treatment arms. p values (from Pearson’s chi-square tests) or 95% CIs are presented where a 10% difference in the percentage of patients was observed either for between-treatment-arm comparisons (p values) or for between-pre- and post-randomization comparisons (95% CIs); on medical review, a difference of 10% was considered clinically meaningful.

### Indirect comparison of ferric citrate and LaC

The median number of sevelamer tablets needed per patient per day to achieve similar regulation of phosphate levels was estimated to be 1.1 and 3.0 for one tablet of ferric citrate and LaC, respectively. This was based on the reported data of a median of 8.0 ferric citrate tablets versus 9.0 sevelamer tablets, and a median of 3.0 LaC tablets and 9.0 sevelamer tablets. Using the mean values, the corresponding values were 2.8 LaC tablets and 9.6 sevelamer tablets, resulting in a dose relativity of 3.4 (Table 3); means were not reported for the ferric citrate data. Using these data, an indirect comparison was made for ferric citrate and LaC. This analysis showed that for every LaC tablet taken, a patient would need to take 2.7 (3.0 using mean values) ferric citrate tablets to achieve similar phosphate levels (Table 3).

**Table 3.** Indirect comparison of ferric citrate and lanthanum carbonate dose relativity and tablet burden using published data^a,b^.

Active treatment	Pill burden per patient per day	Number of sevelamer tablets needed to achieve similar phosphate levels to those of active treatment	Number of sevelamer tablets needed to achieve similar phosphate levels relative to one tablet of active treatment
Ferric citrate^9,c^	8.0 (median)	9.0 (median)	9.0/8.0 = 1.1 tablets
LaC^11,d^	3.0 (median)	9.0 (median)	9.0/3.0 = 3.0 tablets
	2.8 (mean)	9.6 (mean)	9.6/2.8 = 3.4 tablets
Indirect comparison			Relative dosing requirement
Ferric citrate^a^:LaC	−	−	3.0/1.1 = 2.7 tablets (median/median)
			3.4/1.1 = 3.0 tablets (mean/median)

LaC: lanthanum carbonate.

a This indirect comparison used data from two published studies in patients with end-stage renal disease; the first was a randomized controlled trial of ferric citrate versus active control (sevelamer carbonate, calcium acetate or both),^9^ and the second was a real-world evidence study of LaC and sevelamer hydrochloride.^11^ Using these data, tablet burden for each phosphate binder (ferric citrate or LaC) was compared with sevelamer carbonate/hydrochloride. An indirect comparison between ferric citrate and LaC was then performed.

b These comparisons assumed the following: LaC (1000-mg tablet), sevelamer carbonate (800-mg tablet) and sevelamer hydrochloride (800-mg tablet).

c Comparisons for ferric citrate used the median values only, because the mean values were not reported by Lewis et al.^9^ Data from 441 randomized patients (ferric citrate, n = 292; active control, n = 149) from this study were included in this analysis.

d Wilson et al.^11^ presented data from a real-world evidence study of LaC; baseline assessment of phosphate levels was therefore made on patients’ previous phosphate binder (sevelamer hydrochloride) and not at the end of a washout period. Data from 950 patients (who were receiving sevelamer hydrochloride at baseline) from this study were included for this analysis.

## Discussion

Iron overload in patients undergoing haemodialysis is associated with an increased risk of cardiovascular mortality and adverse events.^4,13–16^ Quantitative magnetic resonance imaging (qMRI) is now the gold standard method for the estimation of liver iron concentration and iron store monitoring.^17^ Recent qMRI studies have indicated a substantially higher prevalence of iatrogenic iron overload in patients undergoing haemodialysis than previously established^18,19^ and have raised concerns over guideline biomarker targets for iron store levels. Investigating the risks associated with iron-based versus non-iron-based phosphate binders is clinically relevant and important for patient safety. It was, therefore, important for previously unpublished iron parameter data on patients receiving LaC (a non-iron-based phosphate binder) to be made available for comparison. This analysis has shown that there is no evidence of iron accumulation in patients with ESRD receiving LaC or other non-iron-based phosphate binders for up to 24 months. Comparable results were found in a 6-month comparator study (SPD405-301)^20^ in patients (⩾18 years old) with ESRD who had received haemodialysis for three consecutive months and had hyperphosphataemia (>5.6 mg/dL), which showed no clinically relevant changes in iron parameters or anti-anaemic drug usage between the treatment groups (LaC vs calcium carbonate) after 6 months.^21^ A single-dose randomized study in healthy volunteers has previously reported that calcium-based phosphate binders, but not sevelamer, reduced supplemental iron absorption,^22^ but there is no current evidence to suggest non-supplemental iron accumulation with non-iron-based phosphate binders. Given the concerns about iron overload in patients with ESRD, the results of this study are important for treatment considerations.

Although there was no significant difference in serum ferritin levels between patients receiving LaC and those receiving ST in SPD405-307, an overall increase was observed in both groups from the baseline to month 24/final visit. This increase could potentially be explained by the higher percentage of patients receiving intravenous iron post-randomization, which was likely to be due to patients seeing their nephrologist more frequently because of enrolment in this study. Intravenous iron is associated with elevated serum ferritin, but dosing changes in other concomitant medications, such as erythropoiesis-stimulating agents, are also thought to impact ferritin levels.^23^ This is in contrast to the study by Lewis et al.,^9^ which found significant increases in ferritin and TSAT levels in patients receiving ferric citrate compared with active control (calcium acetate and/or sevelamer carbonate) after 52 weeks.

Our indirect comparison of LaC and ferric citrate highlighted the large number of ferric citrate tablets required to achieve similar phosphate binding to that seen with LaC. Ferric citrate contains 210 mg of elemental iron per tablet,^6^ which equals 567.0 mg (630.0 mg based on mean values) of iron for the 2.7 (3.0 based on mean values) ferric citrate tablets needed to achieve a phosphate-binding dose similar to that of LaC; this is above the daily dose of elemental iron typically used to treat iron deficiency in adults and patients with chronic kidney disease (150−200 mg).^24,25^

One of the main limitations of this study is that although serum ferritin and TSAT measurements are the recommended iron indices by the National Kidney Foundation Kidney Disease Outcome Quality Initiative’s guidelines,^3^ they are indirect indicators of iron status and as such could potentially produce inaccurate values for patients on dialysis.^26^ Furthermore, long-term studies are required to understand fully the potential risks associated with iron overload in patients with ESRD receiving specific iron-based phosphate binders. Phosphate binders have been developed and licenced to treat hyperphosphataemia in patients with ESRD. In these patients, iron neutrality is the desired effect, and any need for iron supplementation should be addressed with appropriate treatments.^3^ An additional limitation of this study was that the tablet burden considerations were based on an indirect comparison of published data. However, for the indirect comparison, both trials used a bioequivalent control (sevelamer carbonate or hydrochloride), and phosphate levels after titration remained consistent over time in all the treatment and control arms for both studies,^9,11^ enabling the comparison to be performed. A direct comparison, although not available, would give a more comprehensive assessment of comparative tablet burden.

## Conclusion

In conclusion, this analysis of iron parameter data from a phase 3 trial shows that there is no evidence of iron accumulation in patients with ESRD treated with LaC or with other non-iron-based phosphate binders.
